# The Alteration of m^6^A Modification at the Transcriptome-Wide Level in Human Villi During Spontaneous Abortion in the First Trimester

**DOI:** 10.3389/fgene.2022.861853

**Published:** 2022-06-08

**Authors:** Jiajie She, Kaifen Tan, Jie Liu, Shuo Cao, Zengguang Li, You Peng, Zhuoyu Xiao, Ruiying Diao, Liping Wang

**Affiliations:** ^1^ The First Affiliated Hospital of Shenzhen University, Reproductive Medicine Centre, Shenzhen Second People’s Hospital, Shenzhen, China; ^2^ Shenzhen Institutes of Advanced Technology, Chinese Academy of Sciences, Shenzhen, China; ^3^ Department of Developmental Biology, School of Basic Medical Sciences, Southern Medical University, Guangzhou, China; ^4^ Department of Obstetrics and Gynecology, Nanfang Hospital, Southern Medical University, Guangzhou, China

**Keywords:** N6 methyladenine (m^6^A), spontaneous abortion, early pregnancy, MeRIP-seq, villous tissues

## Abstract

A growing number of studies have demonstrated that N6 methyladenine (m^6^A) acts as an important role in the pathogenesis of reproductive diseases. Therefore, it is essential to profile the genome-wide m^6^A modifications such as in spontaneous abortion. In this study, due to the trace of human villi during early pregnancy, we performed high-throughput sequencing in villous tissues from spontaneous abortion (SA group) and controls with induced abortion (normal group) in the first trimester. Based on meRIP-seq data, 18,568 m^6^A peaks were identified. These m^6^A peaks were mainly located in the coding region near the stop codon and were mainly characterized by AUGGAC and UGGACG motif. Compared with normal group, the SA group had 2,159 significantly upregulated m^6^A peaks and 281 downregulated m^6^A peaks. Biological function analyses revealed that differential m^6^A-modified genes were mainly involved in the Hippo and Wnt signaling pathways. Based on the conjoint analysis of meRIP-seq and RNA-seq data, we identified thirty-five genes with differentially methylated m^6^A peaks and synchronously differential expression. And these genes were mainly involved in the Wnt signaling pathway, phosphatase activity regulation, protein phosphatase inhibitor activity, and transcription inhibitor activity. This study is the first to profile the transcriptome-wide m^6^A methylome in spontaneous abortion during early pregnancy, which provide novel insights into the pathogenesis and treatment of spontaneous abortion in the first trimester.

## Introduction

Spontaneous abortion (SA) is considered to be one of the most common and severe complications during early pregnancy, which affects 10–15% of pregnant women (Rossen et al., 2018). The etiology of SA is multifactorial, which mainly includes endocrine abnormalities, immune abnormalities, abnormal uterine anatomy, prethrombotic state, chromosome abnormality and infection factors. There may still be other unknown factors contributing to SA, so further investigation is needed. To date, the role of some epigenetic modifications (DNA methylation, histone modification, and non-coding RNA) in SA has been well identified (Liu et al., 2018; Wang et al., 2019; Chen et al., 2021). As the most abundant epigenetic modification of mRNA in eukaryotic cells, m^6^A modification affects the stability (Wang et al., 2014; Ke et al., 2017; Huang et al., 2020), translation (Meyer et al., 2015; Wang et al., 2015; Shi et al., 2017), and splicing of mRNA (Xiao et al., 2016; Pendleton et al., 2017). Although previous studies have also illuminated that m^6^A modification plays important roles in the regulation of immune function and inflammatory response, the relationship between m^6^A methylation and SA remains to be elucidated.

As known, the m^6^A modification includes three main components: 1) “writers”, the methyltransferase complex, such as METTL3 (methyltransferase-like 3), METTL14 (methyltransferase-like 14) (Wang et al., 2016), and METTL16 (methyltransferase-like 16) (Mendel et al., 2018); 2) ‘readers’, RNA binding proteins, including YTHDF1/2/3 (YTH-family proteins 1/2/3), and YTHDC1/2 (YTH domain containing proteins 1/2) (Xu et al., 2015; Gao et al., 2019), and IGF2BP1/2/3 (insulin-like growth factor 2 mRNA binding proteins 1/2/3) (Huang et al., 2020); 3) “erasers”, demethylases, including ALKBH5 (alkB homolog 5) (Zheng et al., 2013) and FTO (fat mass and obesity-associated protein) (Jia et al., 2011). Recently, studies have shown that YTHDF2 can regulate oocyte maturation in female mice and negatively regulate the JAK-STAT pathway to affect the development of mouse nervous system (Ivanova et al., 2017). Wang et al. found that FTO can inhibit the expression level of myogenin, and thus inhibit the differentiation of muscle cells. Li et al. found that ALKBH5 might inhibit the invasion of trophoblast cells in patients with recurrent pregnancy loss, inhibit trophoblast invasion and thus affect mRNA stability. Furthermore, m^6^A modification has also been confirmed to be associated with embryonic stem cell differentiation (Aguilo et al., 2015; Geula et al., 2015), hematopoietic system development (Zhang et al., 2017), myogenesis (Wang et al., 2017), and early embryonic development (Sui et al., 2020). Xia’s lab mapped the transcriptome-wide m^6^A profiles of major fetal tissues including human placenta (Xiao et al., 2019), but the m^6^A methylome in human villi during spontaneous abortion in the first trimester has not been characterized.

In this study, due to the trace of early villous tissue, we performed high-throughput sequencing to determine the transcriptome-wide m^6^A methylome in human villi from patients with spontaneous abortion and controls with induced abortion in the first trimester. Using MeRIP-seq data, we further identified differential m^6^A peaks in villous tissue based on the comparison of spontaneous abortion and induced abortion samples. Then, we identified differentially expressed genes using RNA-seq data. Finally, the conjoint analysis of MeRIP-seq and RNA-seq revealed some genes with differentially methylated m^6^A peaks and synchronously differential expression, which might provide an alternative strategy for the therapy and prevention of spontaneous abortion.

## Materials and Methods

### Samples Collection

Villous tissues from patients with spontaneous abortion and controls with induced abortion used in this research were obtained with written informed consent from all participants. All tissues were approved by the Medicine Ethics Committee of Shenzhen Second People’s Hospital (Approval number, 20210517001-FS01). The inclusion criteria were: (1) Patients who were clinically diagnosed with spontaneous abortion for the first time or healthy women who underwent voluntary induced abortion; (2) Age between 18 and 35; (3) Gestational ages between 6-8 weeks. The exclusion criteria were: (1) Patients with fetal chromosomal or congenital abnormalities; (2) Patients with abnormal uterine structures; (3) Patients with polycystic ovary syndrome, endometriosis, and thyroid disease; (4) Patients with vaginitis. Three spontaneous abortion samples and three induced abortion samples were obtained from elective terminations of apparently normal pregnancies. These samples were further used for MeRIP-seq. The villous tissue was rinsed in precooled normal saline three times, while the tissue with a diameter of 1 mm was cut with ophthalmic scissors. After the tissue was mixed in Trizol solution in a volume ratio of 1:10, it was ground to homogenate with a freeze grinder, and the left tissue was frozen in liquid nitrogen.

### MeRIP Sequencing

Total RNA from each sample was isolated using TRIzol reagent (Invitrogen) and fragmented into ∼100-nucleotide-long fragments by zinc acetate. Next, Affinity-purified anti-m^6^A polyclonal antibodies (Abcam) were used for immunoprecipitation to analyze approximately 300 μg of fragmented RNA. After stringent washing with a high-salt buffer (400 mM NaCl, 0.05% NP-40, 10 mM Tris–HCl), competitive buffer (150 mM NaCl, 0.05% NP-40, 10 mM Tris–HCl, 0.25 mg ml−1 mix of adenosine, uridine, guanosine and cytidine), high-detergent buffer (150 mM NaCl, 0.5% NP-40,10 mM Tris–HCl), and immunoprecipitation buffer (150 mM NaCl, 0.05% NP-40, 10 mM Tris–HCl), bound RNA was eluted by competition with 1 mg ml−1 N6-methyladenosine (Selleckchem) and used for library construction using the NEBNext Ultra RNA Library Prep Kit v2 for Illumina. After removal of ribosomal RNA using the Epicentre Ribo-zero rRNA Removal Kit (Epicentre), total RNA from each tissue was fragmented and a library was constructed using the NEBNext Ultra RNA Library Prep Kit v2 for Illumina (New England Biolabs) as input RNA. RNA-seq libraries of m^6^A antibody-enriched mRNAs and input mRNAs were prepared. Sequencing was carried out using an Illumina Hiseq 4000 platform according to the manufacturer’s instructions.

### Data Analysis

Trimmomatic (v.0.27) (Bolger et al., 2014) was used for quality control of paired-end sequencing data. Reads that mapped to rRNA and tRNA sequences (obtained from the UCSC gene annotation (hg38)) using bowtie2 (v.2.3.4) (Langmead and Salzberg, 2013) were discarded, and the remaining reads were aligned to the GRCh38 using hisat2-align (v.2.1.0) (Kim et al., 2015). Unique reads with high mapping quality were retained using Picard (v.2.16.0) and SAMtools (v.1.7.0). MACS2 (v.2.1.1) (Gaspar, 2018) was used to identify m^6^A peaks with the parameter ‘--nomodel’ and ‘-q 0.05’. ExomePeak2 package (v.1.2.0) (Meng et al., 2014) was used for the identification of differentially methylated peaks. The GFF annotation file was referred to determine the strand information of m^6^A peaks. The findMotifsGenome.pl Perl script from the Homer software suite was used for motif search with the “-mask -rna -len 6” parameters. Genomic locations were split into CDS, 5′UTR, 3′UTR, promoter (2 kb upstream and 100 bp downstream of the TSS), and intergenic regions. The distribution of m^6^A peaks on mRNA was analyzed using the R package Guitar (v.1.7.0) (Cui et al., 2016). Differentially expressed genes were identified using the RNA-seq data (the corresponding MeRIP-seq input library data) by the R package DEseq2 (v.1.32.0) (Love et al., 2014). The R package clusterProfiler (v.4.1.3) (Wu et al., 2021) was used to calculate the biological significance of differentially methylated genes and differentially expressed genes through the Gene Ontology (GO) database and the latest Kyoto Encyclopedia of Genes and Genomes (KEGG) database.

### The Construction of Hub Gene Network

The STRING (v11.5) (https://string-db.org/cgi/input.pl) (Szklarczyk et al., 2021) has been widely applied to construct a protein-protein interaction (PPI) network. Based on those DEGs, the “Multiple proteins” option was selected. The minimum required interaction score was set as “high confidence (0.700)” and a PPI network was constructed. And then, the cytoHubba (Chin et al., 2014) was employed to identify hub genes. The eccentricity algorithm was selected and twenty top-ranked genes were chosen as hub genes. Finally, Cytoscape (v3.9.0) (Demchak et al., 2014) was used to visualize the hub gene network.

### Statistical Analyses

The *t*-test was used for comparing the statistical significance between two groups. For each analysis, *p* < 0.05 was considered as statistically significant.

### Data Availability 

The raw sequencing and processed data reported in this study have been deposited in the Sequence Read Archive (SRA) and Gene Expression Omnibus (GEO) database and are accessible at https://dataview.ncbi.nlm.nih.gov/object/PRJNA786693 and https://www.ncbi.nlm.nih.gov/geo/query/acc.cgi?acc=GSE193052, respectively.

## Results

### Profiles of m^6^A Modification in Human Villi With Spontaneous Abortion in the First Trimester

**FIGURE 1:**
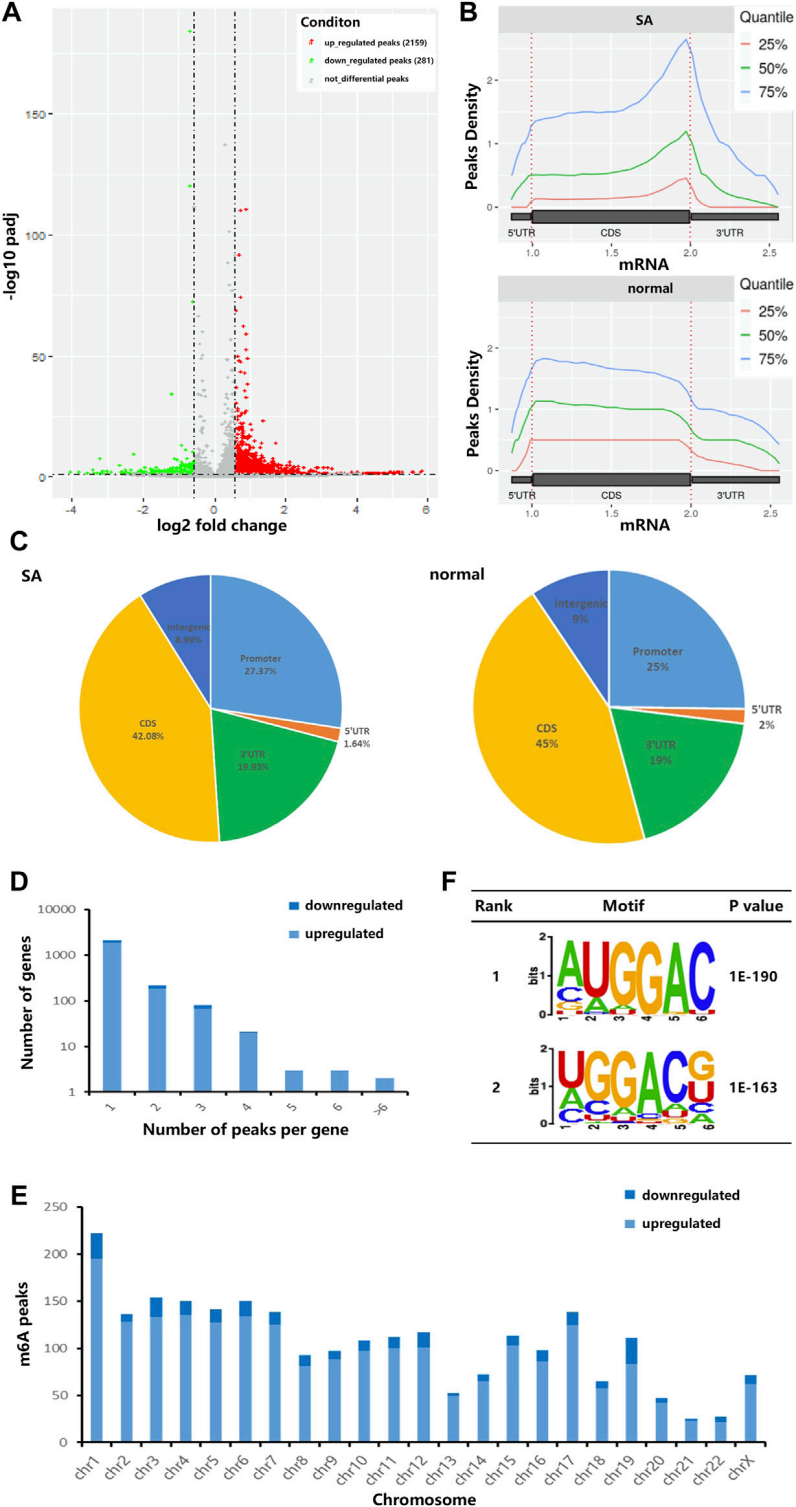
Characteristics of m^6^A methylation in human villi during spontaneous abortion in the first trimester. **(A)** Volcano plots showing the significantly altered m^6^A peaks. **(B)** Accumulation of the region of average m^6^A peaks along with all transcripts in SA group and normal group. **(C)** Pie charts showing the distribution of m^6^A peaks in SA group and normal group. **(D)** The distribution of altered m^6^A peaks per gene. **(E)** The distributions of altered m^6^A peaks in all chromosomes. **(F)** The top two m^6^A motifs enriched from the altered m^6^A peaks.

Due to the trace of early villous tissue, we performed a micro MeRIP-seq analysis of villous tissues from three patients with spontaneous abortion (SA group) and three controls with induced abortion (normal group) in the first trimester. We detected a total of 18,568 m^6^A peaks in these two groups by R package exomePeak2. As shown in Figure 1A, compared with normal group, SA group had 2,159 significantly upregulated m^6^A peaks, which corresponded to transcripts of 2,087 genes, and 281 significantly downregulated m^6^A peaks, which corresponded to transcripts of 311 genes (| log2 (fold change) | > 0.585 and *p* < 0.05). The top 20 altered m^6^A peaks were listed in Table 1. Then, we investigated the distribution of m^6^A peaks in the SA and normal group and found that m^6^A peaks in the SA and normal group were primarily enriched in the coding sequence (CDS) near the stop codon and the whole CDS region, respectively (Figure 1B). However, m^6^A peaks in the SA group showed a distinct pattern from m^6^A peaks in the normal group with a relative decrease in the number of m^6^A peaks in the coding sequence (CDS) (42.08 *vs*. 45%) and 5′ untranslated region (5′UTR) (1.64 *vs*. 2%) and a relative increase in the 3′ untranslated region (3′UTR) (19.93 *vs*. 19%) (Figure 1C). By analyzing the distribution of m^6^A peaks per gene, we found that most genes only had one corresponding m^6^A peak (1,878/236 genes with upregulated and downregulated peaks, respectively) (Figure 1D). Furthermore, dysregulated m^6^A peaks were found in all chromosomes, except chrY, and were mainly found in chr1, chr2, chr3, chr4, chr5, chr6, chr7, chr17 (Figure 1E). Moreover, the m^6^A peaks were mainly characterized by AUGGAC and UGGACG motif (Figure 1F).

**TABLE 1:** Top 20 altered m^6^A peaks in human villi during spontaneous abortion in the first trimester.

Chr	Peak start	Peak end	Peak region	Gene name	Regulation	Log2(FC)	P value
chr4	158,171,401	158,172,272	intron	FAM198B	up	5.830	5.76E-03
chr4	119,033,176	119,033,301	3′ UTR	MYOZ2	up	5.829	4.81E-03
chr4	453,467	453,542	intron	ABCA11P	up	5.778	8.64E-03
chr1	169,376,663	169,377,063	exon	NME7	up	5.586	8.54E-03
chrX	101,488,487	101,488,537	non-coding	ARMCX4	up	5.580	7.82E-03
chr4	37,020,627	37,022,548	TTS	LOC100508631	up	5.563	8.18E-03
chr16	69,356,783	69,357,056	exon	TMED6	up	5.263	1.13E-02
chr8	18,221,796	18,221,846	5′ UTR	NAT1	up	5.198	1.48E-02
chr9	88,462,616	88,475,585	intron	NXNL2	up	5.165	1.59E-02
chr4	13,615,416	13,615,466	exon	BOD1L1	up	5.157	1.38E-02
chr4	372,742	375,592	3′ UTR	MIR571	down	−4.054	7.14E-03
chr11	78,658,228	78,658,303	exon	NARS2	down	−3.808	1.70E-02
chr16	12,572,889	12,573,014	3′ UTR	MIR4718	down	−3.588	3.27E-02
chr3	37,819,313	37,819,463	3′ UTR	ITGA9-AS1	down	−3.415	1.37E-03
chr7	83,135,139	83,135,189	exon	PCLO	down	−3.388	2.05E-02
chr1	241,595,581	241,595,642	TTS	CHML	down	−3.307	2.46E-02
chr1	147,757,292	147,758,409	3′ UTR	GJA5	down	−3.233	4.10E-08
chrX	72,204,881	72,204,956	3′ UTR	PIN4	down	−3.199	4.94E-02
chr3	37,819,488	37,819,538	3′ UTR	ITGA9-AS1	down	−3.167	2.36E-03
chr22	30,663,347	30,663,422	TTS	DUSP18	down	−3.159	2.39E-02

3′UTR, 3′untranslated region; 5′UTR, 5′untranslated region; TTS, transcription termination site.

### GO and KEGG Pathway Enrichment Analysis of Differentially m^6^A-Modified mRNA

**FIGURE 2:**
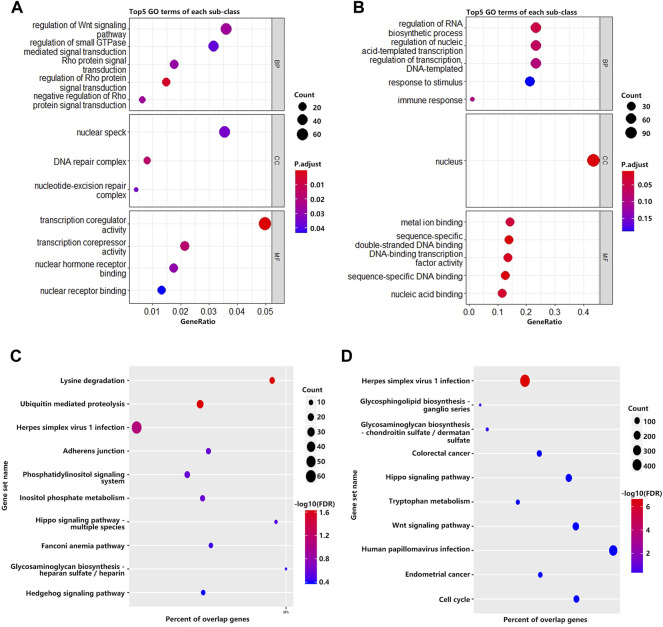
GO and KEGG pathway enrichment analyses of differentially methylated mRNA. **(A)** The top 5 GO terms of genes with significantly upregulated m^6^A peaks. **(B)** The top 5 GO terms of genes with significantly downregulated m^6^A peaks. **(C)** The top 10 KEGG pathways of genes with significantly upregulated m^6^A peaks. **(D)** The top 10 KEGG pathways of genes with significantly downregulated m^6^A peaks.

**FIGURE 3:**
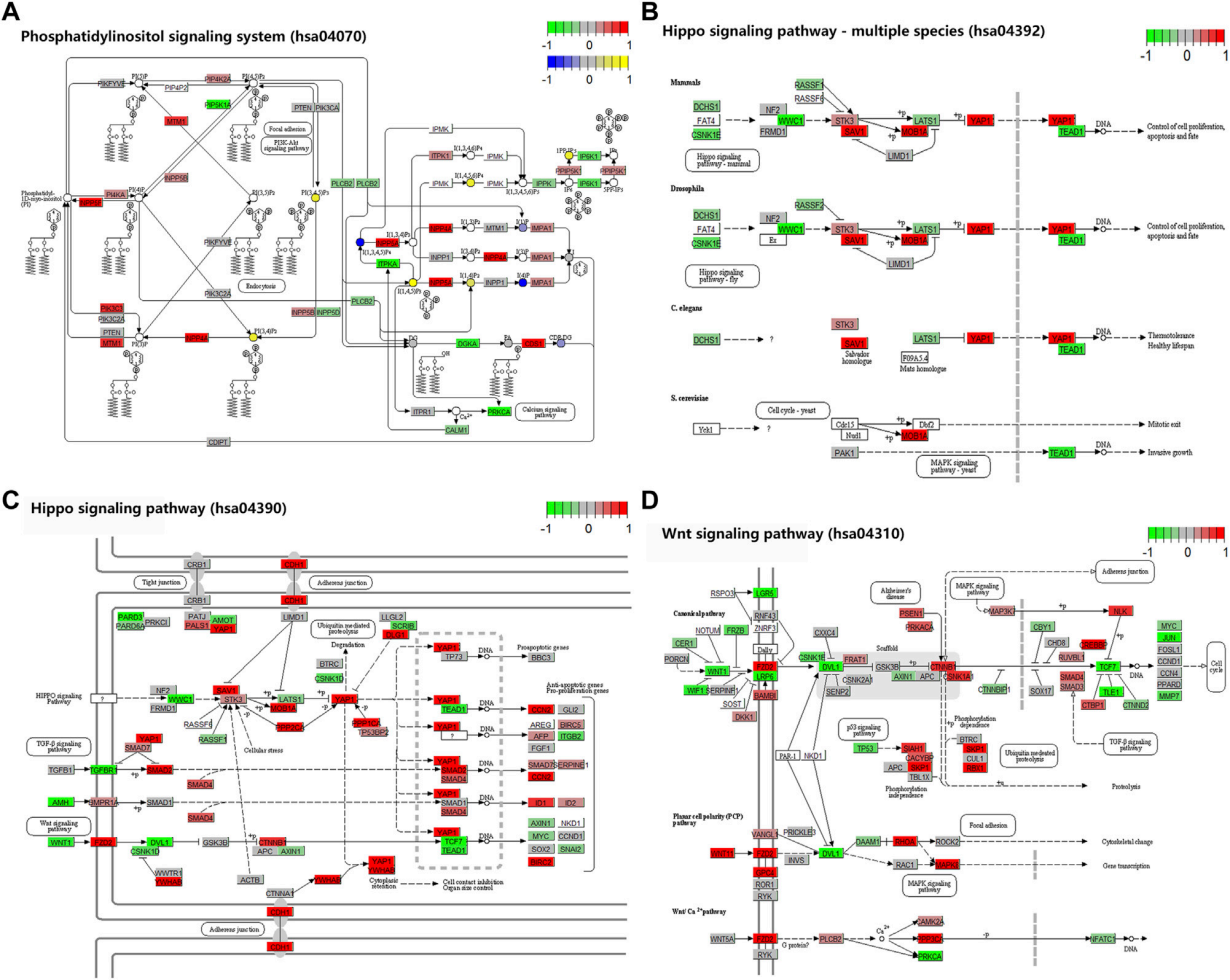
Four major KEGG pathways. **(A)** Phosphatidylinositol signaling system. **(B)** Hippo signaling pathway–multiple species. **(C)** Hippo signaling pathway. **(D)** Wnt signaling pathway. The colors in the graph from red to green indicate the change from high to low in m^6^A dysregulation on genes. The colors in the graph from yellow to blue indicate the change from high to low in m^6^A dysregulation on compounds.

To investigate the biological significance of m^6^A modification in villous tissues of patients with spontaneous abortion and controls with induced abortion in the first trimester, we performed GO and KEGG pathway enrichment analyses of differentially methylated mRNAs. GO ontology was classified into three categories: biological process (BP), cellular component (CC), and molecular function (MF). The top five significantly enriched BPs, CCs, and MFs of genes with upregulated and downregulated m^6^A peaks were shown in Figures 2A,B, respectively. The results in Figure 2A indicated that GO terms such as the regulation of Wnt signaling pathway, Rho protein signal transduction, transcription coregulator activity, and transcription corepressor activity were significantly enriched, and GO terms such as regulation of RNA biosynthetic process, regulation of nucleic acid-templated transcription and DNA-binding transcription factor activity were significantly enriched in Figure 2B. For KEGG pathway enrichment analysis, we found that genes with upregulated m^6^A peaks in villous tissues of patients with spontaneous abortion in the first trimester were significantly associated with the lysine degradation, ubiquitin-mediated proteolysis, herpes simplex virus 1 infection, adherens junction, phosphatidylinositol signaling system, inositol phosphate metabolism, and Hippo signaling pathway - multiple species (Figure 2C). Genes with downregulated m^6^A peaks were significantly associated with herpes simplex virus 1 infection, glycosphingolipid biosynthesis-ganglio series, Hippo signaling pathway, tryptophan metabolism, and Wnt signaling pathway (Figure 2D). The enrichment of genes in the four major pathways is shown in Figure 3.

### Overview of mRNA Expression Profiles and Conjoint Analysis of meRIP-Seq and RNA-Seq

**FIGURE 4:**
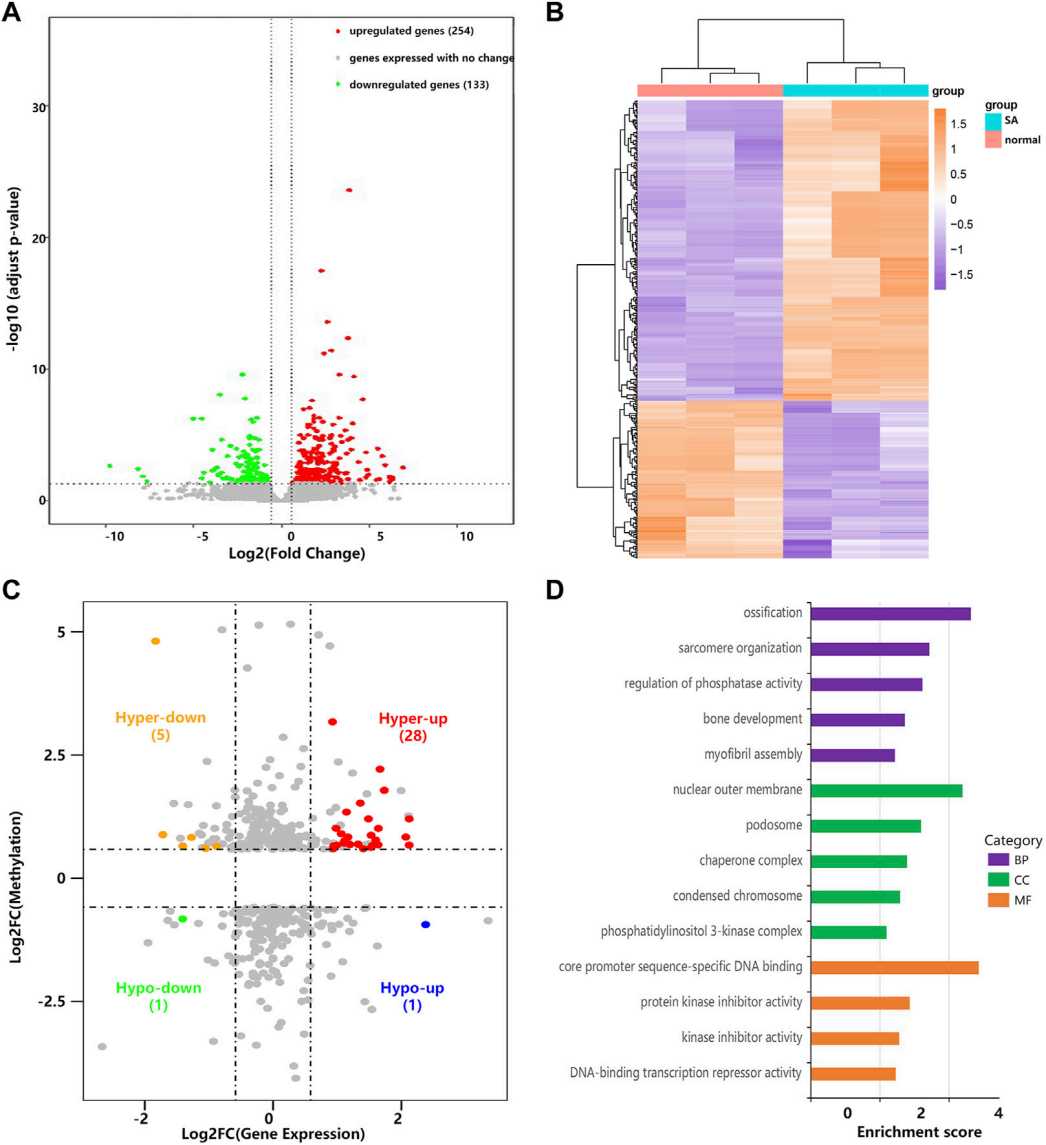
Conjoint analysis of MeRIP-seq and RNA-seq data. **(A)** Volcano plots showing the differentially expressed genes in villous tissues of SA group compared with normal group. **(B)** Heatmap plots showing the differentially expressed genes in villous tissues of SA group compared with normal group. **(C)** Four-quadrant plots showing the distribution of genes with significant changes in both the m^6^A modification and mRNA levels. **(D)** The top 5 GO terms of genes with significant changes in both the m^6^A modification and mRNA levels.

Through RNA-seq (meRIP-seq input library), we detected the transcriptome profiles of villous tissues from patients with spontaneous abortion and controls with induced abortion in the first trimester. R package DESeq2 was used to detect differentially expressed genes (DEGs). Compared with normal group, SA group had 254 significantly upregulated genes and 133 significantly downregulated genes (| log2 (fold change) | > 0.585 and *p* < 0.05; Figures 4A,B). The MA plot was visualized for these DEGs (Supplementary Figure S1). The top 20 DEGs are listed in Table 2. The top 5 significantly enriched BPs, CCs, and MFs of genes with upregulated and downregulated expressed genes and top 10 KEGG pathways were displayed in Supplementary Figure S2. Based on these DEGs, we construct a PPI network through the STRING database. The hub genes selected from the PPI network are visualized in Supplementary Figure S3. According to the eccentricity scores, we identified twenty hub genes with highest confidence scores from the network, and found that most of hub genes were related to immune response and embryonic development. Then, we conducted conjoint analysis of the MeRIP-seq and RNA-seq data and explored the relationship between differential m^6^As and host gene expression level. We found that the expression level of host gene of differential m^6^As was lower in both SA group and normal group compared with constitutive m^6^As (Supplementary Figure S4). Dividing all differentially methylated m^6^A peaks with all differentially expressed mRNAs into four groups (hyper-up, hyper-down, hypo-up, and hypo-down), we identified 34 hypermethylated m^6^A peaks in mRNAs that were significantly upregulated (28; hyper-up) or downregulated (6; hyper-down), while 2 hypomethylated m^6^A peaks in mRNAs that were significantly upregulated (1; hypo-up) or downregulated (1; hypo-down) (Figure 4C, Supplementary Table S1). Finally, we performed GO and KEGG pathway enrichment analysis to explore the biological function of those genes (35) with differentially methylated m^6^A peaks and differential expression. The top 5 significantly enriched BPs, CCs, and MFs indicated that these genes were mainly enriched in the ossification (BP category), nuclear outer membrane (CC category), and core promoter sequence-specific DNA binding (MF) (Figure 4D). However, no KEGG pathways were significantly enriched.

**TABLE 2:** The top 20 differentially expressed mRNAs in human villi during spontaneous abortion in the first trimester.

Gene name	Log2(FC)	Regulation	Location	Strand	P-value
LILRB4	6.994372489	up	chr19:54643889-54670359	+	2.56E-05
KRT6A	6.408677648	up	chr12:52487174-52493257	−	0.000353
TMEM176B	6.402794137	up	chr7:150791285-150801360	−	0.000225
TMEM176A	6.289546685	up	chr7:150800403-150805120	+	0.000221
WNT10A	6.231355116	up	chr2:218880363-218899581	+	0.000217
CTD-2020K17.3	6.217088894	up	chr17:45238028-45241734	−	0.000581
CXCL10	6.158743623	up	chr4:76021117-76023497	−	0.000178
TNC	5.941216251	up	chr9:115019578-115118257	−	1.42E-05
CD300E	5.778269717	up	chr17:74609887-74623738	−	2.10E-06
CXCL9	5.640406283	up	chr4:76001275-76007488	−	0.000375
HBE1	−13.27930895	down	chr11:5268345-5505617	−	1.55E-06
HBZ	−9.7258281	down	chr16:152687-154503	+	1.59E-05
SLC4A1	−8.109924704	down	chr17:44248385-44268141	−	3.30E-05
SPTB	−7.906942374	down	chr14:64746283-64879883	−	0.000189
COX4I2	−7.653108088	down	chr20:31637888-31645006	+	0.000574
LINC02484	−4.950756206	down	chr4:34120894-34269747	−	7.19E-10
ADAMTS18	−4.478881307	down	chr16:77247813-77435114	−	0.000323
RAPGEF4	−4.44120067	down	chr2:172735274-173052893	+	6.25E-10
GOLGA2P7	−4.061744557	down	chr15:84199311-84230136	−	0.000712
AGTR1	−3.91772292	down	chr3:148697784-148743008	+	4.73E-05

## Discussion

In this study, we performed high-throughput sequencing to reveal the m^6^A transcriptome-wide map in human villi during spontaneous abortion in the first trimester. Using the MeRIP-seq data, we found 2,398 genes corresponding to 2,440 altered m^6^A peaks, which were highly enriched by the m^6^A consensus motif (RRACH). The enrichment was not perfectly overlapping the RRACH motif described in literature, which might be related to the parameters (-mask -rna -len 6) used for motif search. These genes were mainly involved in lysine degradation, Hippo signaling pathway, ubiquitin-mediated proteolysis, and glycosphingolipid and glycosaminoglycan biosynthesis. Through conjoint analysis of meRIP-seq and RNA-seq data, we identified 35 genes with differentially methylated m^6^A peaks and synchronously differential expression, which revealed the relationship between m^6^A methylation and gene expression. These genes were enriched in the Wnt signaling pathway, phosphatase activity regulation, protein phosphatase inhibitor activity, and transcription inhibitor activity. It was recently reported that m^6^A inhibition through targeted strategies was effective in counteracting different diseases, such as myeloid leukaemia. This could provide a background for the development of therapeutics and for further investigations in the future (Bedi et al., 2020; Garbo et al., 2021; Moroz-Omori et al., 2021; Yankova et al., 2021).

The cellular mechanisms underlying SA are the proliferation and apoptosis of cytotrophoblasts and human decidual cells (Cinar et al., 2012). Studies have shown that at 6-8 weeks in the first trimester of pregnancy, the expression level of TIMP-1 (tissue inhibitor of MMP-2) in villous tissue is significantly decreased, which leads to the abnormal invasion of trophoblast cells, and thus leads to spontaneous abortion (Kesanakurti et al., 2013). As reported, the high expression of e-cadherin (E-cad) in villous tissue affects the invasion of trophoblast, making it difficult for placenta implantation, resulting in spontaneous abortion (Li et al., 2017). MiR126, located in the region of epidermal growth factor-like domain 7 (EGFL7), negatively regulates vascular endothelial growth factor (VEGF), which reduces shallow implantation of trophoblasts, and finally leads to spontaneous abortion (Schmidt et al., 2007).

Basing on the MeRIP-seq data, we identified some differentially methylated mRNAs which were closely linked to many important pathways. KEGG pathway enrichment analysis results indicated that genes with upregulated m^6^A modification sites were involved in the regulation of lysine degradation. Some studies have confirmed the relationship between lysine metabolism and early embryo development. Studies illuminated that lysine deprivation during low-protein diets could adversely affect early embryo development (Van Winkle et al., 2020). Lysine was specific to LSD1, a demethylase, which regulated the expression and appropriate timing of key developmental regulators during early embryonic development (Foster et al., 2010). Our results indicated that m^6^A modification might affect early embryonic development by regulating lysine degradation. For these genes with upregulated m^6^A modification sites, another related pathway was Hippo signaling pathway. Hippo signaling plays a critical role in early embryonic development as low Hippo activity is required for trophoblast differentiation and high Hippo activity permits inner cell mass formation (Wu and Guan, 2021). During murine preimplantation embryogenesis, Hippo signaling pathway is known to play a significant role in lineage segregation and henceforth the formation of blastocysts (Sasaki, 2015). Our results suggested that modulating m^6^A modifications of the Hippo signaling pathway might be a possible therapy for in human villi during spontaneous abortion in the first trimester in the future.

In addition, another related pathway was ubiquitin-mediated proteolysis. Studies suggested that ubiquitin-mediated proteolysis could be used to regulate Hippo signaling and thus participate in early embryonic development (Ma et al., 2018). Therefore, m^6^A modification might affect ubiquitin-mediated proteolysis to regulate Hippo signaling and thus regulate early embryonic development. Genes with downregulated m^6^A modification sites were mainly enriched in glycosphingolipid and glycosaminoglycan biosynthesis. Glycosphingolipids (GSLs) were a class of ceramide-based glycolipids essential for embryo development in mammals (Yamashita et al., 1999; Russo et al., 2016), whether glycosaminoglycan (GAG) biosynthetic was important for mouse embryonic stem cells (mESCs) (Nairn et al., 2007). Based on our results, we hypothesized that m^6^A modification might influence the expression level of some genes related to glycosphingolipid and glycosaminoglycan biosynthesis and thus influence embryo development.

Through the conjoint analysis of MeRIP-seq and RNA-seq data, thirty-five genes with differentially methylated m^6^A peaks and synchronously differential expression in spontaneous abortion were discovered (Supplementary Table S1). Among these genes, IGFBP3, C/EBPβ may be regulated by m^6^A modification of mRNAs. IGFBP3 (Insulin-like growth factor binding protein 3) with high expression level suggested betted oocyte maturation and early embryo development (Wang et al., 2006). IGFBP3 is highly expressed in the endometrium and at the maternal-fetal interface, which promoted the matrix metalloproteinases 2 (MMP2) expression and cell migration in both human endometrial stromal cells (HESCs) and primary human decidual stromal cells (HDSCs) (Luo et al., 2020). Combined with our results, m^6^A modification may promote the expression of IGFBP3 and thus promote the high expression of MMP2 and the low expression of its tissue inhibitors TIMP-1, and finally lead to spontaneous abortion. CCAAT/enhancer binding protein β (C/EBPβ) is the earliest marker of enveloping layer (EVL) and is essential for EVL differentiation in zebrafish (Zhang et al., 2021). Studies indicated that C/EBPβ transcription factor could inhibit the mRNA decay of IL-8 and thus repress the inflammatory response (Zhang et al., 2010). Moreover, C/EBPβ is also a biomarker of endometrial receptivity and plays a conserved functional role during embryo implantation (Kannan et al., 2010). Combined with our results, altered m^6^A modification may influence the expression of C/EBPβ, regulate embryo implantation and thus influence early embryo development. However, detailed molecular mechanisms are still unknown and further exploration deserves careful consideration in the future.

## Conclusion

Here, we systematically investigated the whole-transcriptome m^6^A profile of human villous tissues during spontaneous abortion in the first trimester, revealing a dynamic m^6^A methylation landscape in spontaneous abortion for the first time. Based on the conjoint analysis of MeRIP-seq and RNA-seq data, many genes with differentially methylated m^6^A peaks and synchronously differential expression were discovered. It indicated a potential link between m^6^A methylation and mRNA expression, and might provide an alternative therapeutic strategy for spontaneous abortion. In addition, the m^6^A modification profile might provide novel insights into the pathogenesis and treatment of spontaneous abortion during early pregnancy.

## Data Availability

The original contributions presented in the study are publicly available. This data can be found here: https://www.ncbi.nlm.nih.gov/Traces/study/?acc=SRP350117&o=acc_s%3

## References

[B1] Aguilo F. Zhang F. Sancho A. Fidalgo M. Di Cecilia S. Vashisht A. (2015). Coordination of m(6)A mRNA Methylation and Gene Transcription by ZFP217 Regulates Pluripotency and Reprogramming. Cell Stem Cell 17, 689–704. 10.1016/j.stem.2015.09.005 26526723 PMC4671830

[B2] Bedi R. K. Huang D. Eberle S. A. Wiedmer L. Śledź P. Caflisch A. (2020). Small‐Molecule Inhibitors of METTL3, the Major Human Epitranscriptomic Writer. ChemMedChem 15, 744–748. 10.1002/cmdc.202000011 32159918

[B3] Bolger A. M. Lohse M. Usadel B. (2014). Trimmomatic: A Flexible Trimmer for Illumina Sequence Data. Bioinformatics 30, 2114–2120. 10.1093/bioinformatics/btu170 24695404 PMC4103590

[B4] Chen X. Guo D.-Y. Yin T.-L. Yang J. (2021). Non-Coding RNAs Regulate Placental Trophoblast Function and Participate in Recurrent Abortion. Front. Pharmacol. 12, 646521. 10.3389/fphar.2021.646521 33967782 PMC8100504

[B5] Chin C.-H. Chen S.-H. Wu H.-H. Ho C.-W. Ko M.-T. Lin C.-Y. (2014). cytoHubba: Identifying Hub Objects and Sub-Networks from Complex Interactome. BMC Syst. Biol. 8, S11. 10.1186/1752-0509-8-S4-S11 25521941 PMC4290687

[B6] Cinar O. Kara F. Can A. (2012). Potential Role of Decidual Apoptosis in the Pathogenesis of Miscarriages. Gynecol. Endocrinol. 28, 382–385. 10.3109/09513590.2011.633127 22103816

[B7] Cui X. Wei Z. Zhang L. Liu H. Sun L. Zhang S.-W. (2016). Guitar: An R/Bioconductor Package for Gene Annotation Guided Transcriptomic Analysis of RNA-Related Genomic Features. BioMed Res. Int. 2016, 8367534. 10.1155/2016/8367534 27239475 PMC4864564

[B8] Demchak B. Hull T. Reich M. Liefeld T. Smoot M. Ideker T. (2014). Cytoscape: The Network Visualization Tool for GenomeSpace Workflows. F1000Res 3, 151. 10.12688/f1000research.4492.2 25165537 PMC4133763

[B9] Foster C. T. Dovey O. M. Lezina L. Luo J. L. Gant T. W. Barlev N. (2010). Lysine-Specific Demethylase 1 Regulates the Embryonic Transcriptome and CoREST Stability. Mol. Cell. Biol. 30, 4851–4863. 10.1128/mcb.00521-10 20713442 PMC2950538

[B10] Gao Y. Pei G. Li D. Li R. Shao Y. Zhang Q. C. (2019). Multivalent m6A Motifs Promote Phase Separation of YTHDF Proteins. Cell Res. 29, 767–769. 10.1038/s41422-019-0210-3 31388144 PMC6796879

[B11] Garbo S. Zwergel C. Battistelli C. (2021). m6A RNA Methylation and Beyond - The Epigenetic Machinery and Potential Treatment Options. Drug Discov. Today 26, 2559–2574. 10.1016/j.drudis.2021.06.004 34126238

[B12] Gaspar J. M. (2018). Improved Peak-Calling with MACS2. bioRxiv. 10.1101/496521

[B13] Geula S. Moshitch-Moshkovitz S. Dominissini D. Mansour A. A. Kol N. Salmon-Divon M. (2015). m6A mRNA Methylation Facilitates Resolution of Naïve Pluripotency Toward Differentiation. Science 347, 1002–1006. 10.1126/science.1261417 25569111

[B14] Huang H. Weng H. Sun W. Qin X. Shi H. Wu H. (2020). Publisher Correction: Recognition of RNA N6-Methyladenosine by IGF2BP Proteins Enhances mRNA Stability and Translation. Nat. Cell Biol. 22, 1288. 10.1038/s41556-020-00580-y 32855523

[B15] Ivanova I. Much C. Di Giacomo M. Azzi C. Morgan M. Moreira P. N. (2017). The RNA m6A Reader YTHDF2 Is Essential for the Post-Transcriptional Regulation of the Maternal Transcriptome and Oocyte Competence. Mol. Cell. 67, 1059–1067. 10.1016/j.molcel.2017.08.003 28867294 PMC5613143

[B16] Jia G. Fu Y. Zhao X. Dai Q. Zheng G. Yang Y. (2011). N6-Methyladenosine in Nuclear RNA is a Major Substrate of the Obesity-Associated FTO. Nat. Chem. Biol. 7, 885–887. 10.1038/nchembio.687 22002720 PMC3218240

[B17] Kannan A. Fazleabas A. T. Bagchi I. C. Bagchi M. K. (2010). The Transcription Factor C/EBPβ is a Marker of Uterine Receptivity and Expressed at the Implantation Site in the Primate. Reprod. Sci. 17, 434–443. 10.1177/1933719110361384 20224108 PMC3048823

[B18] Ke S. Pandya-Jones A. Saito Y. Fak J. J. Vågbø C. B. Geula S. (2017). m6A mRNA Modifications are Deposited in Nascent Pre-mRNA and are not Required for Splicing but Do Specify Cytoplasmic Turnover. Genes Dev. 31, 990–1006. 10.1101/gad.301036.117 28637692 PMC5495127

[B19] Kesanakurti D. Chetty C. Dinh D. H. Gujrati M. Rao J. S. (2013). Role of MMP-2 in the Regulation of IL-6/Stat3 Survival Signaling via Interaction with α5β1 Integrin in Glioma. Oncogene 32, 327–340. 10.1038/onc.2012.52 22349830 PMC3368064

[B20] Kim D. Langmead B. Salzberg S. L. (2015). HISAT: A Fast Spliced Aligner with Low Memory Requirements. Nat. Methods 12 (4), 357–360. 10.1038/nmeth.3317 25751142 PMC4655817

[B21] Langmead B. Salzberg S. (2013). Fast Gapped-Read Alignment with Bowtie 2. Nat. Methods 9 (4), 357–359. 10.1038/nmeth.1923 PMC3322381 22388286

[B22] Li P. Shi Y. Shuai H. Cai Y. Lu W. Wang G. (2017). Alterted SLIT2/ROBO1 Signalling is Linked to Impaired Placentation of Missed and Threatened Miscarriage in Early Pregnancy. Histopathology 71, 543–552. 10.1111/his.13250 28485101

[B23] Liu Y. Tang Y. Ye D. Ma W. Feng S. Li X. (2018). Impact of Abnormal DNA Methylation of Imprinted Loci on Human Spontaneous Abortion. Reprod. Sci. 25, 131–139. 10.1177/1933719117704906 28443481

[B24] Love M. I. Huber W. Anders S. (2014). Moderated Estimation of Fold Change and Dispersion for RNA-Seq Data With DESeq2. Genome Biology 15, 550. 10.1186/s13059-014-0550-8 25516281 PMC4302049

[B25] Luo J. Zhu H. Chang H. M. Lin Y. M. Yang J. Leung P. C. K. (2020). The Regulation of IGFBP3 by BMP2 Has a Role in Human Endometrial Remodeling. FASEB J. 34, 15462–15479. 10.1096/fj.202000508R 32975335

[B26] Ma X. Guo X. Richardson H. E. Xu T. Xue L. (2018). POSH Regulates Hippo Signaling Through Ubiquitin-Mediated Expanded Degradation. Proc. Natl. Acad. Sci. U.S.A. 115, 2150–2155. 10.1073/pnas.1715165115 29440430 PMC5834682

[B27] Mendel M. Chen K.-M. Homolka D. Gos P. Pandey R. R. McCarthy A. A. (2018). Methylation of Structured RNA by the m6A Writer METTL16 Is Essential for Mouse Embryonic Development. Mol. Cell 71, 986–1000. 10.1016/j.molcel.2018.08.004 30197299 PMC6162343

[B28] Meng J. Lu Z. Liu H. Zhang L. Zhang S. Chen Y. (2014). A Protocol for RNA Methylation Differential Analysis with MeRIP-Seq Data and exomePeak R/Bioconductor Package. Methods 69, 274–281. 10.1016/j.ymeth.2014.06.008 24979058 PMC4194139

[B29] Meyer K. D. Patil D. P. Zhou J. Zinoviev A. Skabkin M. A. Elemento O. (2015). 5′ UTR m6A Promotes Cap-Independent Translation. Cell 163, 999–1010. 10.1016/j.cell.2015.10.012 26593424 PMC4695625

[B30] Moroz‐Omori E. V. Huang D. Kumar Bedi R. Cheriyamkunnel S. J. Bochenkova E. Dolbois A. (2021). METTL3 Inhibitors for Epitranscriptomic Modulation of Cellular Processes. ChemMedChem 16, 3035–3043. 10.1002/cmdc.202100291 34237194 PMC8518639

[B31] Nairn A. V. Kinoshita-Toyoda A. Toyoda H. Xie J. Harris K. Dalton S. (2007). Glycomics of Proteoglycan Biosynthesis in Murine Embryonic Stem Cell Differentiation. J. Proteome Res. 6, 4374–4387. 10.1021/pr070446f 17915907 PMC3973744

[B32] Pendleton K. E. Chen B. Liu K. Hunter O. V. Xie Y. Tu B. P. (2017). The U6 snRNA m6A Methyltransferase METTL16 Regulates SAM Synthetase Intron Retention. Cell 169, 824–835. 10.1016/j.cell.2017.05.003 28525753 PMC5502809

[B33] Rossen L. M. Ahrens K. A. Branum A. M. (2018). Trends in Risk of Pregnancy Loss Among US Women, 1990-2011. Paediatr. Perinat. Epidemiol. 32, 19–29. 10.1111/ppe.12417 29053188 PMC5771868

[B34] Russo D. Parashuraman S. D’Angelo G. (2016). Glycosphingolipid-Protein Interaction in Signal Transduction. Int. J. Mol. Sci. 17, 1732. 10.3390/ijms17101732 PMC5085762 27754465

[B35] Sasaki H. (2015). Position- and Polarity-Dependent Hippo Signaling Regulates Cell Fates in Preimplantation Mouse Embryos. Semin. Cell Dev. Biol. 47-48, 80–87. 10.1016/j.semcdb.2015.05.003 25986053

[B36] Schmidt M. Paes K. De Mazière A. Smyczek T. Yang S. Gray A. (2007). EGFL7 Regulates the Collective Migration of Endothelial Cells by Restricting Their Spatial Distribution. Development 134, 2913–2923. 10.1242/dev.002576 17626061

[B37] Shi H. Wang X. Lu Z. Zhao B. S. Ma H. Hsu P. J. (2017). YTHDF3 Facilitates Translation and Decay of N6-Methyladenosine-Modified RNA. Cell Res. 27, 315–328. 10.1038/cr.2017.15 28106072 PMC5339834

[B38] Sui X. Hu Y. Ren C. Cao Q. Zhou S. Cao Y. (2020). METTL3-Mediated m6A Is Required for Murine Oocyte Maturation and Maternal-To-Zygotic Transition. Cell Cycle 19, 391–404. 10.1080/15384101.2019.1711324 31916488 PMC7100890

[B39] Szklarczyk D. Gable A. L. Nastou K. C. Lyon D. Kirsch R. Pyysalo S. (2021). The STRING Database in 2021: Customizable Protein-Protein Networks, and Functional Characterization of User-Uploaded Gene/Measurement Sets. Nucleic Acids Res. 49, D605–D612. 10.1093/nar/gkaa1074 33237311 PMC7779004

[B40] Van Winkle L. J. Galat V. Iannaccone P. M. (2020). Lysine Deprivation During Maternal Consumption of Low-Protein Diets Could Adversely Affect Early Embryo Development and Health in Adulthood. Int. J. Environ. Res. Public Health 17, 5462. 10.3390/ijerph17155462 PMC7432313 32751190

[B41] Wang J. Yang J. Yan Y. Zhu Z. Mu Y. Wang X. (2019). Effect of Adoptive Transfer of CD4+CD25+Foxp3+ Treg Induced by Trichostatin A on the Prevention of Spontaneous Abortion. J. Reprod. Immunol. 131, 30–35. 10.1016/j.jri.2018.12.002 30634133

[B42] Wang T. Chang C. Wu H. Chiu Y. Chen C. Wang H. (2006). Insulin-Like Growth Factor-II (IGF-II), IGF-Binding Protein-3 (IGFBP-3), and IGFBP-4 in Follicular Fluid are Associated with Oocyte Maturation and Embryo Development. Fertil. Steril. 86, 1392–1401. 10.1016/j.fertnstert.2006.03.064 17070193

[B43] Wang X. Feng J. Xue Y. Guan Z. Zhang D. Liu Z. (2016). Structural Basis of N^6^-Adenosine Methylation by the METTL3–METTL14 Complex Chemical Modifications of RNA Have Essential Roles in a Vast Range of Cellular Processes. Nature 534 (7608), 575–578. 10.1038/nature18298 27281194

[B44] Wang X. Huang N. Yang M. Wei D. Tai H. Han X. (2017). FTO is Required for Myogenesis by Positively Regulating mTOR-PGC-1α Pathway-Mediated Mitochondria Biogenesis. Cell Death Dis. 8, e2702. 10.1038/cddis.2017.122 28333151 PMC5386528

[B45] Wang X. Lu Z. Gomez A. Hon G. C. Yue Y. Han D. (2014). N^6^-Methyladenosine-Dependent Regulation of Messenger RNA Stability. Nature 505, 117–120. 10.1038/nature12730 24284625 PMC3877715

[B46] Wang X. Zhao B. S. Roundtree I. A. Lu Z. Han D. Ma H. (2015). N^6^-Methyladenosine Modulates Messenger RNA Translation Efficiency. Cell 161, 1388–1399. 10.1016/j.cell.2015.05.014 26046440 PMC4825696

[B47] Wu T. Hu E. Xu S. Chen M. Guo P. Dai Z. (2021). ClusterProfiler 4.0: A Universal Enrichment Tool for Interpreting Omics Data. Innovation(China) 2, 100141. 10.1016/j.xinn.2021.100141 PMC8454663 34557778

[B48] Wu Z. Guan K.-L. (2021). Hippo Signaling in Embryogenesis and Development. Trends Biochem. Sci. 46, 51–63. 10.1016/j.tibs.2020.08.008 32928629 PMC7749079

[B49] Xiao S. Cao S. Huang Q. Xia L. Deng M. Yang M. (2019). The RNA N^6^-Methyladenosine Modification Landscape of Human Fetal Tissues. Nat. Cell Biol. 21, 651–661. 10.1038/s41556-019-0315-4 31036937

[B50] Xiao W. Adhikari S. Dahal U. Chen Y.-S. Hao Y.-J. Sun B.-F. (2016). Nuclear m6A Reader YTHDC1 Regulates mRNA Splicing. Mol. Cell 61, 507–519. 10.1016/j.molcel.2016.01.012 26876937

[B51] Xu C. Liu K. Ahmed H. Loppnau P. Schapira M. Min J. (2015). Structural Basis for the Discriminative Recognition of N^6^-Methyladenosine RNA by the Human YT521-B Homology Domain Family of Proteins. J. Biol. Chem. 290, 24902–24913. 10.1074/jbc.M115.680389 26318451 PMC4598999

[B52] Yamashita T. Wada R. Sasaki T. Deng C. Bierfreund U. Sandhoff K. (1999). A Vital Role for Glycosphingolipid Synthesis During Development and Differentiation. Proc. Natl. Acad. Sci. U.S.A. 96, 9142–9147. 10.1073/pnas.96.16.9142 10430909 PMC17746

[B53] Yankova E. Blackaby W. Albertella M. Rak J. De Braekeleer E. Tsagkogeorga G. (2021). Small-Molecule Inhibition of METTL3 as a Strategy Against Myeloid Leukaemia. Nature 593, 597–601. 10.1038/s41586-021-03536-w 33902106 PMC7613134

[B54] Zhang C. Chen Y. Sun B. Wang L. Yang Y. Ma D. (2017). M6A Modulates Haematopoietic Stem and Progenitor Cell Specification. Nature 549, 273–276. 10.1038/nature23883 28869969

[B55] Zhang X. Wu Z. Bu M. Hu R. Zhang X. Li W. (2021). The CCAAT/Enhancer Binding Protein Beta (Cebpb) Is Essential for the Development of Enveloping Layer (EVL) in Zebrafish. Aquac. Fish. 9 (5), e95087. 10.1016/j.aaf.2021

[B56] Zhang Z. Xing X. Hensley G. Chang L.-W. Liao W. Abu-Amer Y. (2010). Resistin Induces Expression of Pro-Inflammatory Cytokines and Chemokines in Human Articular Chondrocytes via Transcription and mRNA Stabilization. Arthritis Rheum. 62 (7), 1993–2003. 10.1002/art.27473 20506172 PMC3979471

[B57] Zheng G. Dahl J. A. Niu Y. Fedorcsak P. Huang C.-M. Li C. J. (2013). ALKBH5 is a Mammalian RNA Demethylase that Impacts RNA Metabolism and Mouse Fertility. Mol. Cell 49, 18–29. 10.1016/j.molcel.2012.10.015 23177736 PMC3646334

