# Supplementary material for: The Alteration of m6A Modification at the Transcriptome-Wide Level in Human Villi During Spontaneous Abortion in the First Trimester
Source: Front Genet. 2022 Jun 8;13:861853. doi: 10.3389/fgene.2022.861853 (PMC9215105; doi:10.3389/fgene.2022.861853)
Supplement: Supplementary file 2 [file DataSheet1.DOCX]

Supplementary Material

**The alteration of m^6^A modification at the transcriptome-wide level** **in** **human villi during spontaneous abortion**

**in the first trimester**

Jiajie She^1,4†^, Kaifen Tan^2†^, Jie Liu^3†^, Shuo Cao^2^, Shan Xiao^2^, Zengguang Li^2^, You Peng^2^, Zhuoyu Xiao^2^, Ruiying Diao^1*^ and Liping Wang^1*^

†These authors contributed equally to this work.

*To whom correspondence should be addressed.

***Correspondence:** Liping Wang: <wlp18665070696@163.com>; Ruiying Diao: <15889753127@163.com>

# Supplementary Figures and Tables

## Supplementary Figures


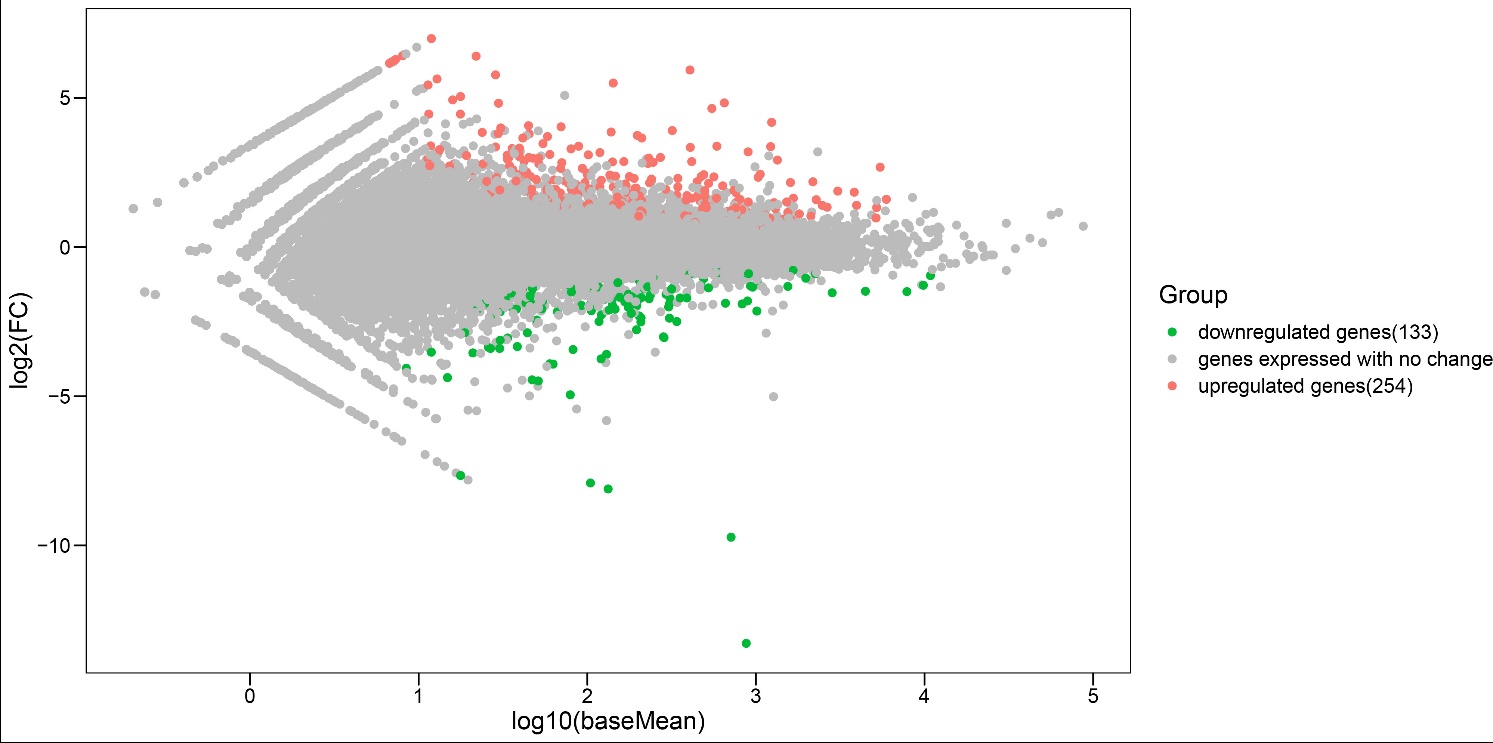


**Supplementary Figure 1. MA plot for differentially expressed genes in villous tissues of SA group compared with normal group.**


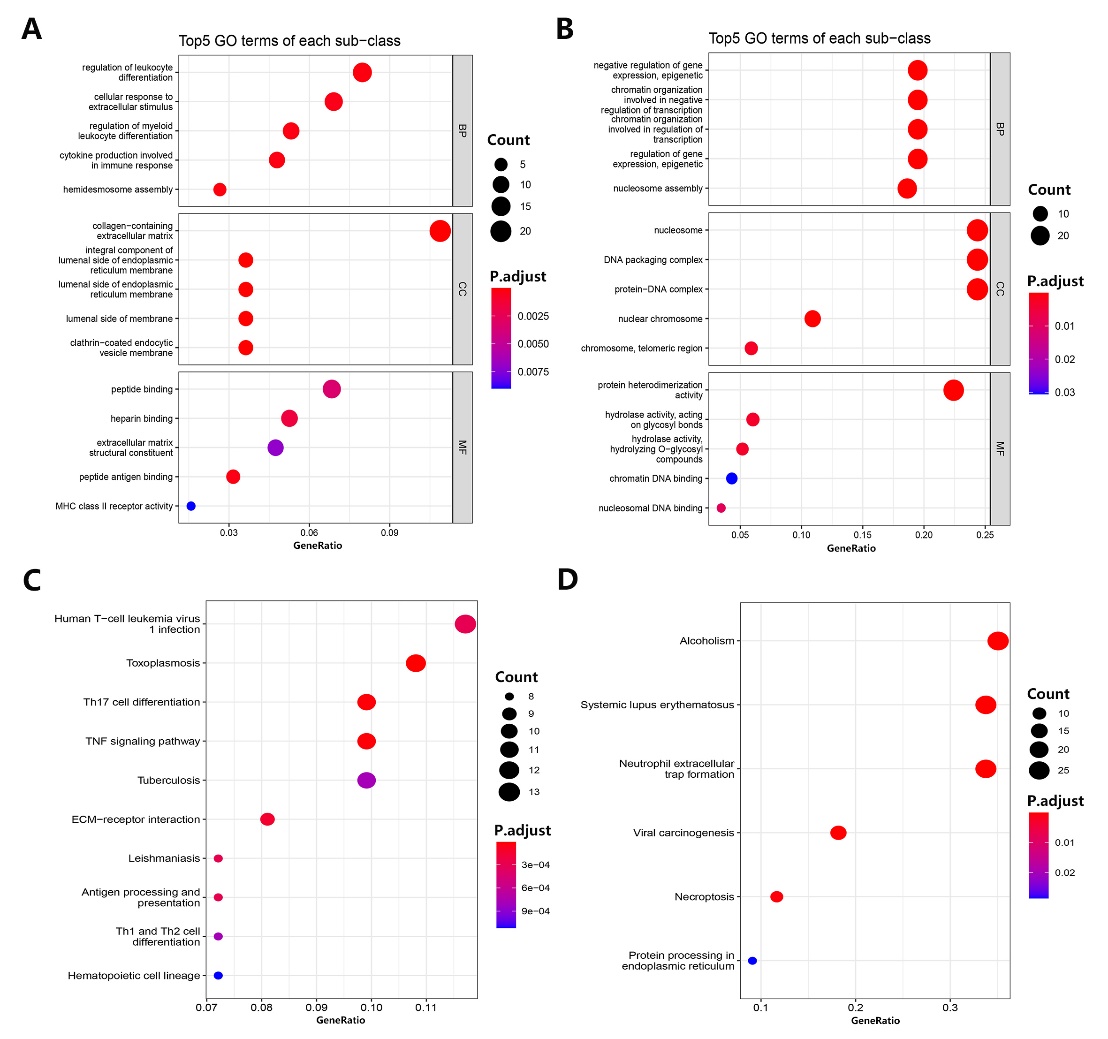


**Supplementary Figure 2. GO and KEGG pathway enrichment analysis of differentially expressed genes.**

(**A**) The top 5 GO terms of upregulated expressed genes. (**B**) The top 5 GO terms of downregulated expressed genes. (**C**) The top 10 KEGG pathways of upregulated expressed genes. (**D**) The top 10 KEGG pathways of downregulated expressed genes.

**
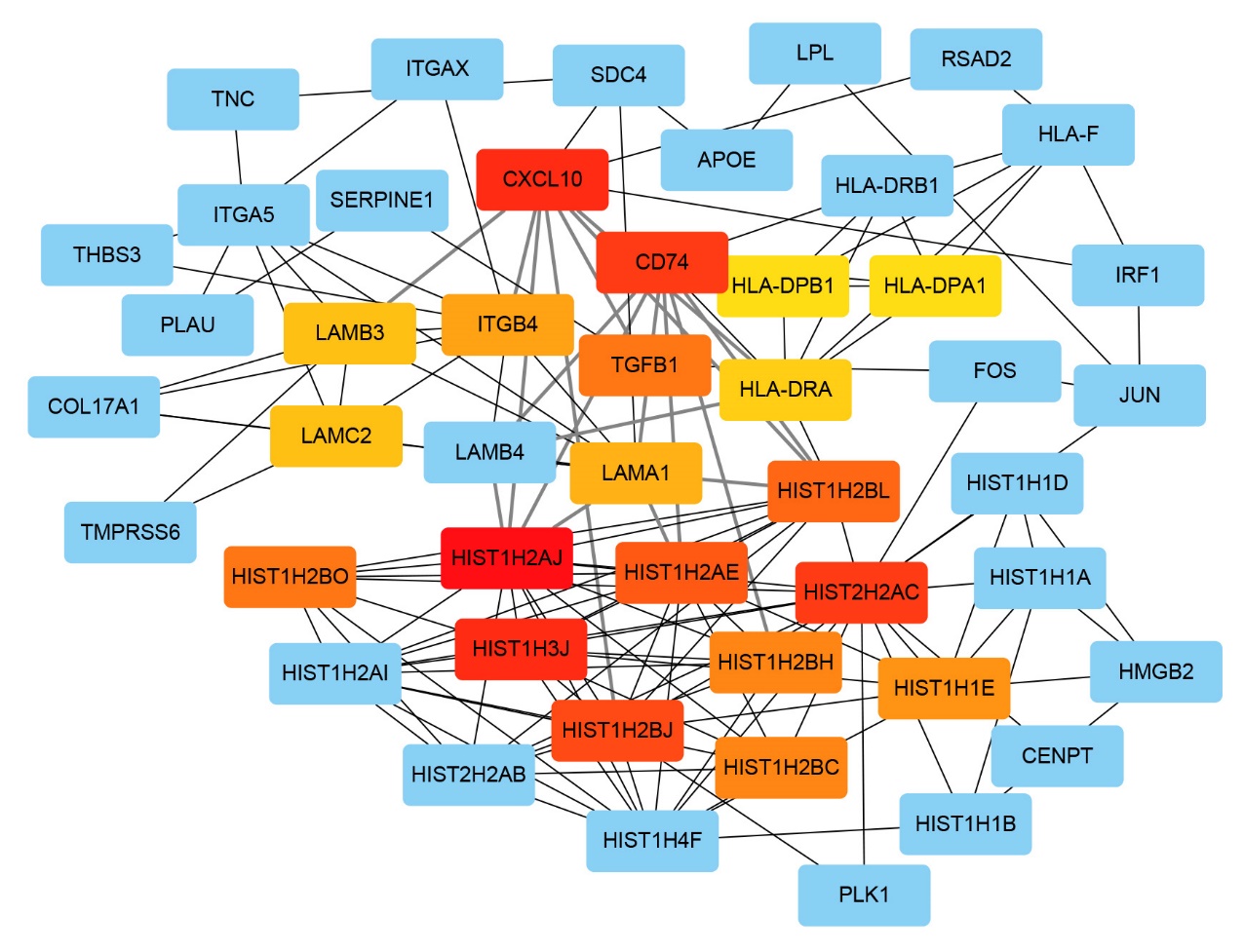
**

**Supplementary Figure 3. Twenty hub genes-based PPI network.**

Twenty hub genes with the most substantial interactions according to the calculated results by eccentricity method. The darker the color of nodes, the higher the score.


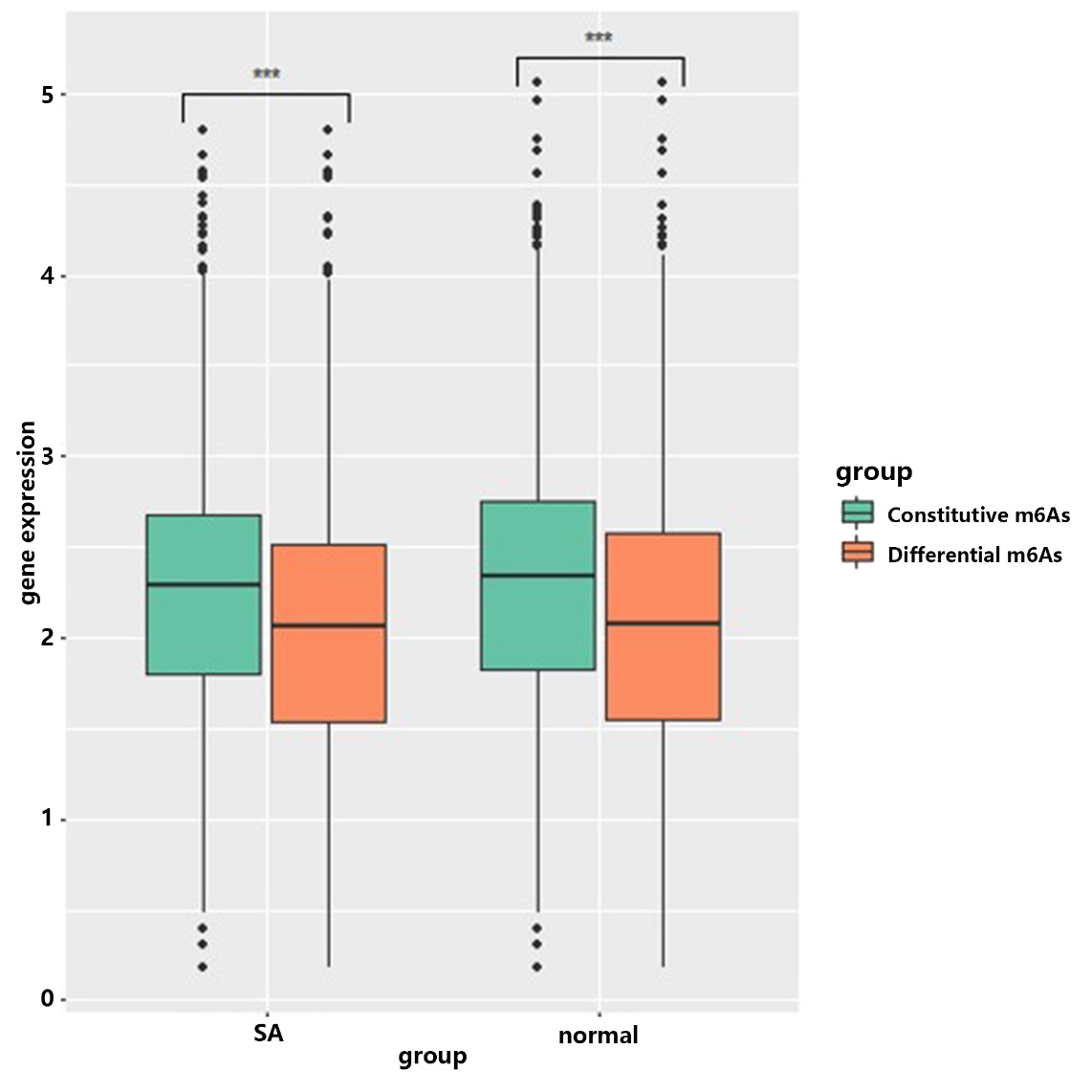


**Supplementary Figure 4. Box plot of the expression level of host gene in SA and normal group.**

The host gene expression level of differential m6As was significantly lower than that of constitutive m6As. Mann Whitney U test, * * * P < 0.001

## Supplementary Tables

Table S1. 35 genes with differentially methylated m6A peaks and synchronously differential expression in spontaneous abortion. (Table S1.xlsx)
